# ^18^F-FIMP: a LAT1-specific PET probe for discrimination between tumor tissue and inflammation

**DOI:** 10.1038/s41598-019-52270-x

**Published:** 2019-10-31

**Authors:** Satoshi Nozaki, Yuka Nakatani, Aya Mawatari, Nina Shibata, William E. Hume, Emi Hayashinaka, Yasuhiro Wada, Hisashi Doi, Yasuyoshi Watanabe

**Affiliations:** 1 Laboratory for Pathophysiological and Health Science, RIKEN Center for Biosystems Dynamics Research and Center for Life Science Technologies, Kobe, Hyogo 650-0047 Japan; 2 Novel PET Diagnostics Laboratory, RIKEN Innovation Center, Hyogo, 650-0047 Japan; 3 Laboratory for Labeling Chemistry, RIKEN Center for Biosystems Dynamics Research and Center for Life Science Technologies, Kobe, Hyogo 650-0047 Japan

**Keywords:** Cancer imaging, Diagnostic markers

## Abstract

Positron emission tomography (PET) imaging can assist in the early-phase diagnostic and therapeutic evaluation of tumors. Here, we report the radiosynthesis, small animal PET imaging, and biological evaluation of a L-type amino acid transporter 1 (LAT1)-specific PET probe, ^18^F-FIMP. This probe demonstrates increased tumor specificity, compared to existing tumor-specific PET probes (^18^F-FET, ^11^C-MET, and ^18^F-FDG). Evaluation of probes by *in vivo* PET imaging, ^18^F-FIMP showed intense accumulation in LAT1-positive tumor tissues, but not in inflamed lesions, whereas intense accumulation of ^18^F-FDG was observed in both tumor tissues and in inflamed lesions. Metabolite analysis showed that ^18^F-FIMP was stable in liver microsomes, and mice tissues (plasma, urine, liver, pancreas, and tumor). Investigation of the protein incorporation of ^18^F-FIMP showed that it was not incorporated into protein. Furthermore, the expected mean absorbed dose of ^18^F-FIMP in humans was comparable or slightly higher than that of ^18^F-FDG and indicated that ^18^F-FIMP may be a safe PET probe for use in humans. ^18^F-FIMP may provide improved specificity for tumor diagnosis, compared to ^18^F-FDG, ^18^F-FET, and ^11^C-MET. This probe may be suitable for PET imaging for glioblastoma and the early-phase monitoring of cancer therapy outcomes.

## Introduction

Positron emission tomography (PET) imaging can assist in early-phase clinical evaluations of tumors. 2-Deoxy-2-^18^F-fluoro-D-glucose (FDG) is the most commonly used PET probe for tumor imaging; however, this probe has several limitations. ^18^F-FDG is actively transported into cells via glucose transporters (GLUTs), which are expressed not only in tumor tissues, but also in inflamed lesions in which they regulate glucose metabolism to facilitate the inflammatory response^1^. Hence, ^18^F-FDG PET cannot distinguish between tumor tissue and inflamed lesions^2^, and therefore results in false-positive results for tumor diagnosis^3^.

Radiolabeled amino acid PET probes, such as L-[methyl-^11^C]-methionine (MET), were developed to overcome the disadvantages of ^18^F-FDG^4^. ^11^C-MET was predicted to have a higher specificity for tumors; however, this probe was found to accumulate in tumor, normal, and inflamed tissues^5,6^. In order to improve the selectivity for tumor tissues, PET probes with tumor-specific molecular targets have developed^7–9^.

The L-type amino acid transporter 1 (LAT1) is a sodium-independent L-type amino acid transporter (LAT), with four isoforms: LAT1, LAT2, LAT3, and LAT4^10^. Since LAT1 is highly expressed in various human tumors it presents a promising target for both imaging and therapeutics^11^. Several PET probes targeting LAT1 have been reported, including ^18^F-fluoro-ethyl-tyrosine (FET)^7^, and L-3-^18^F-fluoro-α-methyl-tyrosine (FAMT)^9^. However, none of these LAT1 probes have been widely used owing to the poor signal/noise ratios^12^. Therefore in order to improve the tyrosine based LAT1 probes, we designed and synthesized various α-methyl-L-phenylalanine derivatives to specifically target LAT1. Among these, the radiolabeled compound (*S*)-2-amino-3-[3-(2-^18^F-fluoroethoxy)-4-iodophenyl]-2-methylpropanoic acid (^18^F-FIMP; Development code: AA-7), was identified as a potentially effective PET probe for LAT1. Here, we report the radiosynthesis, small animal PET imaging, and biological evaluation of ^18^F-FIMP.

## Results

As a result of screening our α-methyl amino acid chemical library using hLAT1 and hLAT2 overexpression cell lines, FIMP was found to have higher affinity for LAT1 than 2-aminobicyclo-(2,2,1)-heptane-2-carboxylic acid (BCH), a classical inhibitor of L-type amino acid transporters, which is also transported into cells as a substrate of LAT1. The half-maximal (50%) inhibitory concentration (IC50) value of FIMP was significantly lower than that of BCH (Mean ± SD, 88.5 ± 13.5 µM and 231.5 ± 10.0 µM, respectively).

### Expression of human LAT1 and CD98 in cell lines and tumors

**Figure 1:**
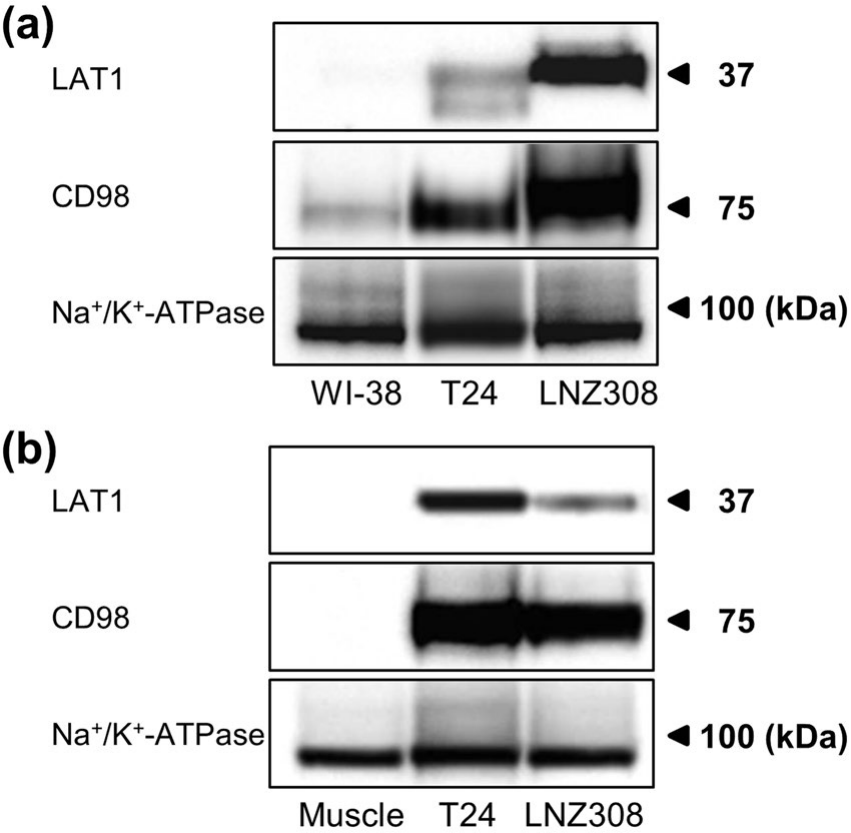
Expression of human LAT1 and CD98 in human tumor cell lines and tumors. Western blot analyses were performed using the anti-human LAT1 and CD98 antibodies on membrane protein extracts from three human cell lines (WI-38, T24 and LNZ308) and three mice tissues (muscle, T24 and LNZ308 xenografts). The same blots were also probed with anti-Na+/K+-ATPase antibody as a loading control. Full-length uncropped blots are included in Supplementary Figs S2 and S3.

High expression levels of LAT1 and CD98 were observed for T24 and LNZ308 tumor cells. Conversely, WI-38 normal human fetal lung fibroblast showed low to moderate expression of both proteins (Fig. 1a).

We also evaluated LAT1 and CD98 expression in tumor xenografts and normal muscle tissue. Moderate to high LAT1 and CD98 expressions were observed for T24 and LNZ308 xenografts (Fig. 1b). Conversely, LAT1 and CD98 expression was not detected for normal muscle tissue. Expression of sodium/potassium ATPase, plasma membrane markers, was comparable in all cells and tissues (Methods in Supplementary Information).

### PET probe accumulation in tumor and inflamed tissue

**Figure 2:**
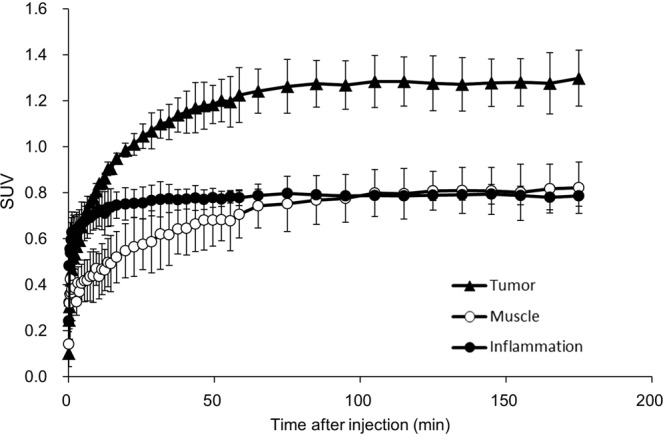
Time-activity curve of ^18^F-FIMP. Time-activity curves of ^18^F-FIMP accumulation in the tumor (filled triangle), muscle (open circle) and inflamed tissue (filled circle) over a 180 minute dynamic PET scan. Data are expressed as mean SUV ± SD (N = 3).

**Figure 3:**
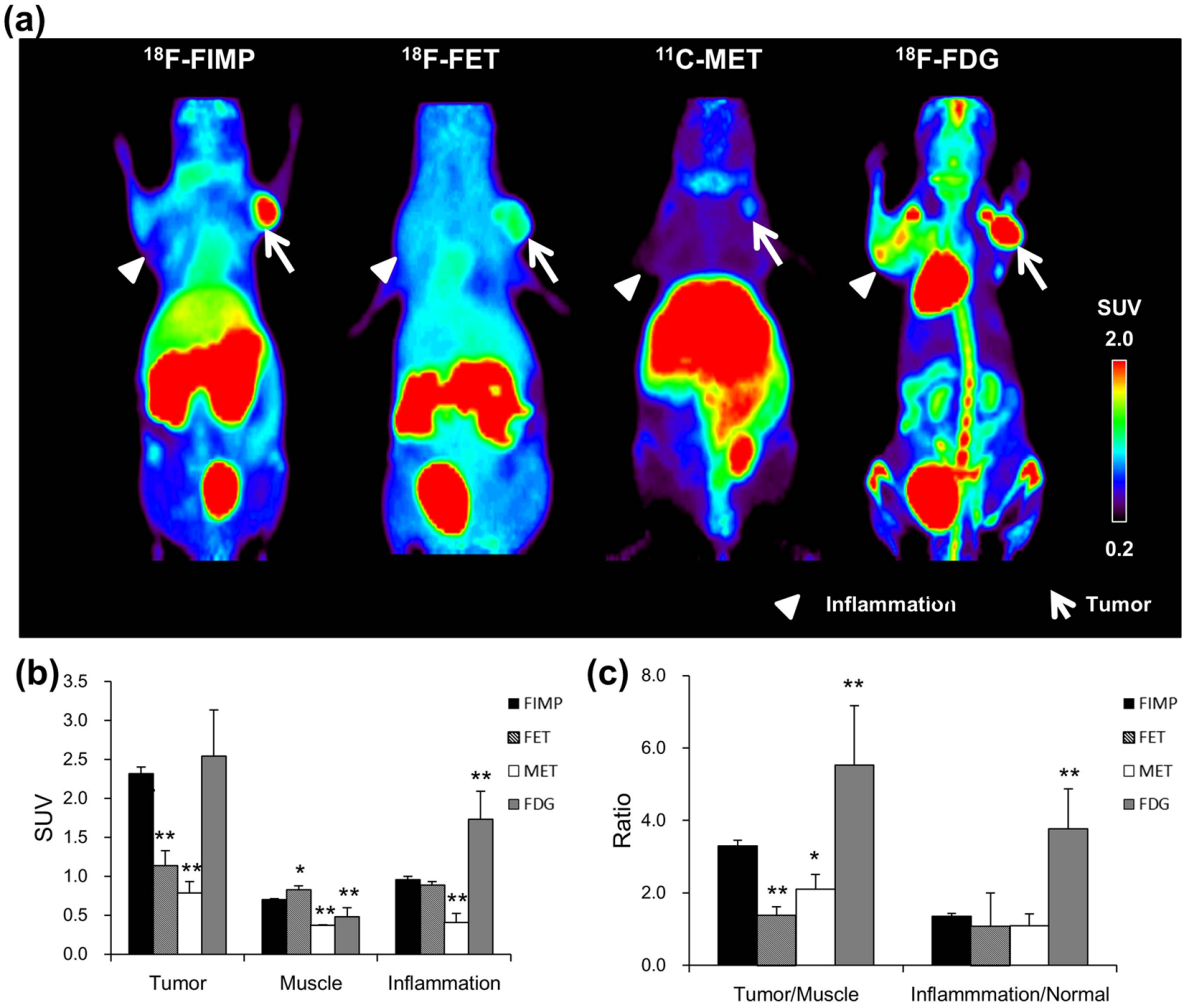
PET probe accumulation in tumor and inflamed tissue. (**a)** Maximum intensity projection (MIP) image of ^18^F-FIMP, ^18^F-FET, ^11^C-MET, and ^18^F-FDG PET. LNZ308 cells were inoculated in right paws (arrow), and inflammation induced by injection of turpentine oil in left paws (arrow head). PET data were acquired 90 min after injection of ^18^F-FIMP, ^18^F-FET, and ^11^C-MET, ^18^F-FDG-PET data was acquired from 55 to 100 min after injection. Quantitative analysis of PET imaging data represented as **(b)** SUV and **(c)** tumor-to-muscle and inflamed lesion-to-normal muscle ratios (TMR and INR, respectively). Data are presented as mean ± SD (n = 4–9). *P < 0.05, **P < 0.01, compared with ^18^F-FIMP groups.

PET probe accumulations were evaluated by small animal PET imaging using a mouse model which had both acute inflammation and a LAT1-positive tumor. After probe injection we observed time-dependent change of ^18^F-FIMP accumulation by PET imaging in tumor, muscle and inflamed tissues. Time-activity curve of ^18^F-FIMP accumulation in all tissues plateaued by 90 min after probe injection (Fig. 2). On the basis of this result, the biodistribution in all animals was evaluated at 90 min after probe injection. As a result of visual assessment, ^18^F-FIMP showed high accumulation in the tumor region and low accumulation in the inflamed lesion. ^18^F-FDG showed higher accumulation in tumor regions compared to ^18^F-FIMP, ^18^F-FET and ^11^C-MET; however, comparatively high^18^F-FDG accumulation was also observed in inflamed lesions compared to ^18^F-FIMP, ^18^F-FET and ^11^C-MET. ^18^F-FET showed moderate accumulation in tumor regions and low accumulation in inflamed regions. ^11^C-MET showed low accumulation in both tumor regions and inflamed regions (Fig. 3a). Furthermore, quantitative analyses of PET images found that accumulation levels of ^18^F-FIMP in tumors (SUV 2.32 ± 0.09) were significantly higher than that of ^18^F-FET (SUV 1.14 ± 0.20) and ^11^C-MET (SUV 0.79 ± 0.14) (P < 0.01, respectively), comparable to that of ^18^F-FDG (SUV 2.55 ± 0.59) without significant difference (P = 0.29). However, accumulation of ^18^F-FIMP in inflamed lesions (SUV 0.96 ± 0.04) was comparable and significantly higher than that of ^18^F-FET (SUV 1.14 ± 0.20) and ^11^C-MET (SUV 0.79 ± 0.14) (P = 0.19 and P < 0.01, respectively), significantly lower than that of ^18^F-FDG (SUV 1.73 ± 0.36) (P < 0.01) (Fig. 3b).

Calculated tumor-to-muscle and inflamed lesion-to-normal muscle ratios (TMR and INR, respectively) further supported these findings. TMR of ^18^F-FIMP (3.29 ± 0.16) was significantly higher than that of ^18^F-FET (1.38 ± 0.25) and ^11^C-MET (2.11 ± 0.40) (P < 0.01 and P < 0.05, respectively), significantly lower than that of ^18^F-FDG (5.52 ± 1.65) (P < 0.01). INR of ^18^F-FIMP (1.36 ± 0.08) was comparable to that of ^18^F-FET (1.07 ± 0.92) and ^11^C-MET (1.10 ± 0.32) (P = 0.09 and P = 0.23, respectively), significantly lower than that of ^18^F-FDG (3.77 ± 1.10) (P < 0.01) (Fig. 3c). These ratios suggested that ^18^F-FDG accumulated not only in tumor tissues but also in inflamed lesions. Conversely, ^18^F-FIMP accumulation was high in tumor tissues, but low in inflamed lesions.

**Figure 4:**
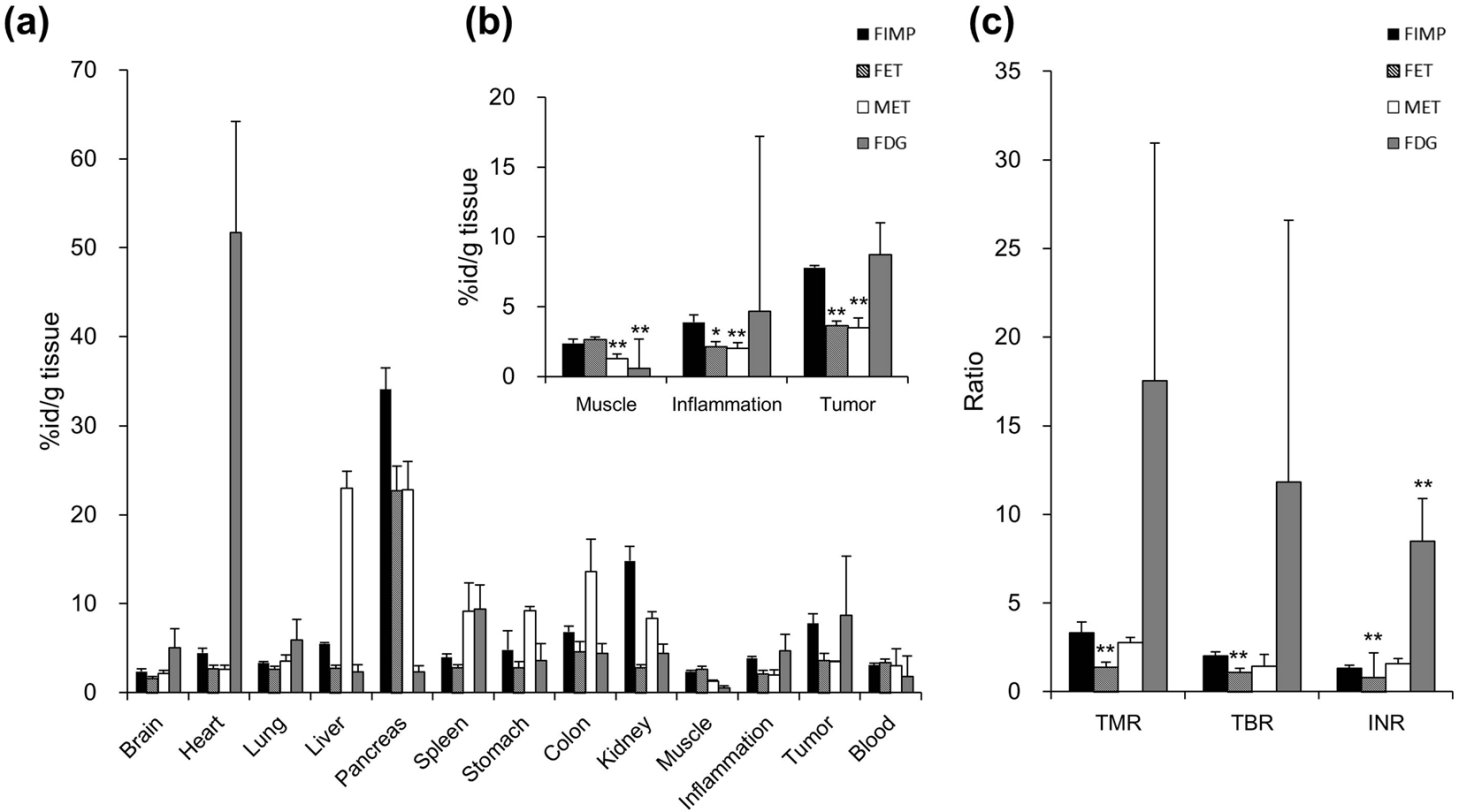
Biodistribution of ^18^F-FIMP in tumors and selected organs. Quantitative analysis of biodistribution data presented as **(a)** percentage of injected activity per gram of tissue (%ID/g), **(b)** %ID/g in muscle, inflamed, and tumor tissue, and **(c)** tumor-to-muscle, tumor-to-blood, inflamed tissue-to-normal muscle ratios (TMR, TBR, and INR, respectively). Data are presented as mean ± SD (n = 6). *P < 0.05, **P < 0.01, compared with ^18^F-FIMP groups.

Tissue radioactivity was measured to validate the PET imaging data. Probe biodistribution data showed highest ^18^F-FIMP uptake in the pancreas (33.99 ± 2.39%ID/g). Uptake of ^18^F-FIMP was higher in tumor (7.78 ± 1.11%ID/g) than inflamed tissue, muscle tissue, and blood (3.89 ± 0.17, 2.36 ± 0.14, and 3.10 ± 0.22%ID/g, respectively). ^18^F-FET uptake was highest in the pancreas (22.73 ± 2.78%ID/g). Tumor uptake of ^18^F-FET (3.64 ± 0.77%ID/g) was higher compared to inflamed tissue and normal muscle, but equivalent to that of blood (2.12 ± 0.37, 2.67 ± 0.27, and 3.37 ± 0.38%ID/g, respectively). ^11^C-MET uptake was highest in the liver and pancreas (23.02 ± 1.91 and 22.84 ± 3.15%ID/g, respectively). Tumor uptake of ^11^C-MET (3.49 ± 0.05%ID/g) was higher compared to inflamed tissue and normal muscle, but equivalent to that of blood (2.00 ± 0.55, 1.27 ± 0.14, and 3.03 ± 1.89%ID/g, respectively). In contrast, ^18^F-FDG uptake was highest in the heart (51.70 ± 12.52%ID/g); however, tumor uptake (8.71 ± 6.61%ID/g) was also higher compared to inflamed tissue, muscle, and blood (4.67 ± 1.91, 0.56 ± 0.19, and 1.79 ± 2.35%ID/g, respectively) (Fig. 4a). ^18^F-FIMP uptake in tumor tissue (7.78 ± 1.11%ID/g) was significantly higher than that of ^18^F-FET (3.64 ± 0.77%ID/g) and ^11^C-MET (3.49 ± 0.05%ID/g) (P < 0.01, respectively), comparable to that of ^18^F-FDG (8.71 ± 6.61%ID/g) without significant difference (P = 0.77). On the other hand, accumulation of ^18^F-FIMP in inflamed lesions (3.89 ± 0.17%ID/g) was significantly higher than that of ^18^F-FET (2.12 ± 0.37%ID/g) and ^11^C-MET (2.00 ± 0.55%ID/g) (P < 0.05 and P < 0.01, respectively), not significantly difference than that of ^18^F-FDG (4.67 ± 1.91%ID/g) (P = 0.14) (Fig. 4b). TMR of ^18^F-FIMP (3.32 ± 0.62) was significantly higher and comparable than that of ^18^F-FET (1.37 ± 0.28) and ^11^C-MET (2.78 ± 0.29) (P < 0.01 and P = 0.17, respectively), not significantly difference than that of ^18^F-FDG (17.5 ± 13.4) (P = 0.08). INR of ^18^F-FIMP (1.32 ± 0.16) was significantly lower and comparable to that of ^18^F-FET (0.80 ± 1.39) and ^11^C-MET (1.57 ± 0.30) (P < 0.01 and P = 0.20, respectively), significantly lower than that of ^18^F-FDG (8.48 ± 2.44) (P < 0.01) (Fig. 4c).

### PET probe accumulation in inflamed joints of Collagen-induced arthritis (CIA) mice

Accumulation of ^18^F-FDG, but not ^18^F-FIMP or ^11^C-MET, was observed in inflamed joints of CIA mice. No accumulation of any probe was detected in the joints of control mice (Fig. S1A in Supplementary Information). Moreover, quantitative analysis from PET data demonstrated that the inflamed joint-to-normal muscle ratio (INR) of ^18^F-FDG was significantly higher for CIA mice (4.66 ± 0.70) compared to control mice (2.31 ± 0.15) (P < 0.001). However, there was no significant difference in INR for ^18^F-FIMP and ^11^C-MET accumulation in CIA mice (0.91 ± 0.01 and 0.68 ± 0.20), compared to controls (0.86 ± 0.04 and 0.68 ± 0.11) (Fig. S1B in Supplementary Information).

### Metabolic stability

**Figure 5:**
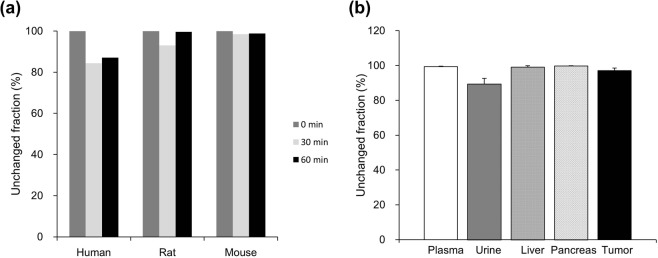
Metabolic stability of ^18^F-FIMP. **(a)** ^18^F-FIMP loading in liver microsomes was measured by LC-MS/MS at 30 and 60 min and the ratio of unmetabolized fraction to the total fraction was determined. Data are presented as mean (n = 2). **(b)** The ratio of radioactivity in the unmetabolized fraction to that in total radioactivity was determined using a phosphoimaging plate at 90 min after injection into tumor-bearing mice. Data are presented as mean ± SD (n = 4).

The metabolic stability of ^18^F-FIMP was evaluated under *in vitro* and *in vivo* conditions (Methods in Supplementary Information). *In vitro* experiments using human, rat, and mouse liver microsomes, showed that FIMP was highly stable over 60 min, and the unchanged fraction were 87.1, 99.6, and 98.8% for human, rat and mouse microsomes, respectively (Fig. 5a). The stability of ^18^F-FIMP was also confirmed *in vivo* using tumor-bearing mice, in which the compound was stable for 90 min following administration. The *in vivo* unchanged fraction at 90 min were 99.4 ± 0.3, 89.4 ± 3.2, 99.0 ± 0.8, 99.8 ± 0.1, and 97.1 ± 1.4% in plasma, urine, liver, pancreas, and tumor tissue, respectively (Fig. 5b).

### Protein incorporation

**Figure 6:**
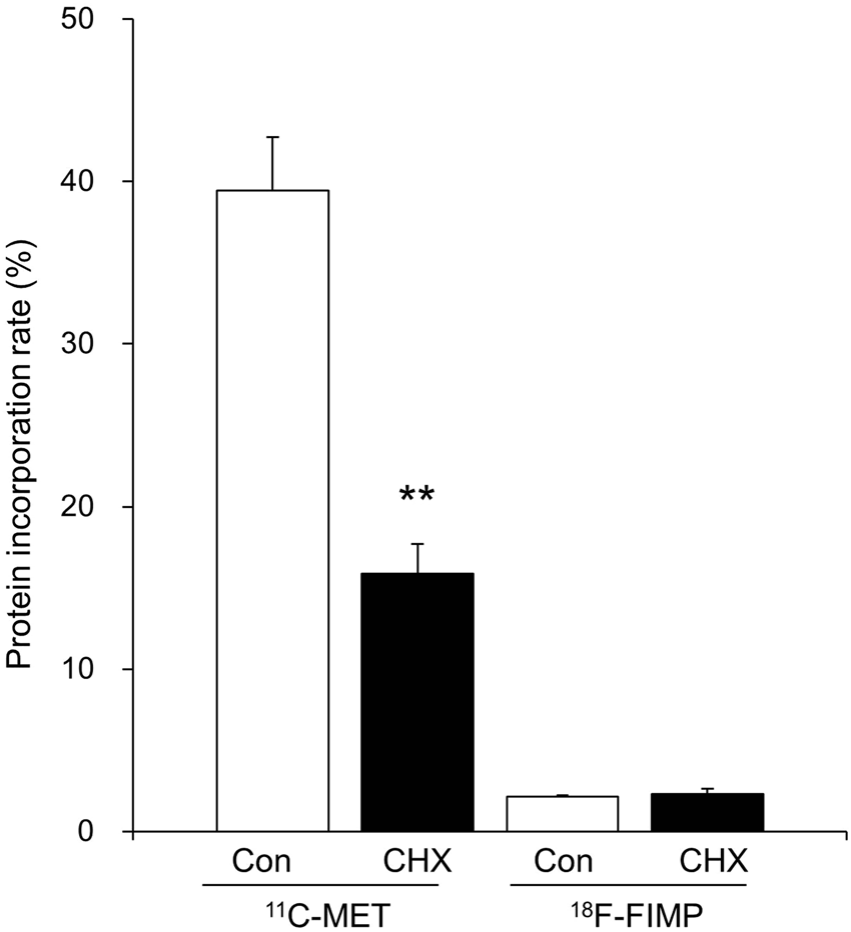
Protein incorporation assay. Incorporation of radioactivity into cell protein fractions with or without cycloheximide (CHX) treatment. Data are presented as mean ± SD (n = 6). *P < 0.05, **P < 0.01, compared with control (untreated, CHX^−^) groups.

^11^C-MET was detected at high levels (39.4 ± 3.3%) in the acid precipitation fraction of LNZ308 cells. This value significantly decreased (15.9 ± 1.8%) with pretreatment with cycloheximide (CHX), a protein synthesis inhibitor, was performed (P < 0.01). However, incorporation of ^18^F-FIMP was very low both with and without CHX pretreatment (2.1 ± 0.1% and 2.3 ± 0.3%; Fig. 6). Furthermore, no significant difference was observed between CHX-treated and untreated cells.

### Dosimetry analysis

The mean absorbed dose of ^18^F-FIMP for humans was estimated according to mouse biodistribution data (Table 1). Absorption was highest in the human pancreas, with absorbed doses of 268.5 ± 137.0 and 299.3 ± 152.0 μSv/MBq for males and females, respectively. Organs showing moderate absorbed doses included the liver (86.3 ± 80.4 and 115.3 ± 103.9 μSv/MBq, for males and females, respectively), kidneys (64.7 ± 26.5 and 71.4 ± 28.9 μSv/MBq), and urinary bladder wall (41.3 ± 7.1 and 53.6 ± 9.9 μSv/MBq). The effective doses were 25.1 ± 5.3 and 30.8 ± 6.7 μSv/MBq for males and females, respectively.

**Table 1:** Predicted human absorbed doses for ^18^F-FIMP (MIRD method).

Organ	Estimated absorbed dose (μSV/MBq)	
	Male	Female
Adrenals	23.7 ± 7.0	29.5 ± 8.7
Brain	9.5 ± 0.4	12.1 ± 0.5
Breasts	10.5 ± 1.4	13.2 ± 1.7
Gallbladder Wall	29.0 ± 13.4	34.7 ± 16.2
Gastrointestinal tract		
Stomach Wall	19.2 ± 2.7	24.1 ± 3.6
Small Intestine	15.8 ± 7.0	18.4 ± 2.5
Upper Large Intestine Wall	16.5 ± 3.1	20.7 ± 3.8
Lower Large Intestine Wall	13.8 ± 0.7	17.5 ± 0.8
Heart Wall	16.7 ± 3.5	21.4 ± 4.9
Kidneys	64.7 ± 26.5	71.4 ± 28.9
Liver	86.3 ± 80.4	115 ± 103
Lungs	14.3 ± 3.2	18.7 ± 4.3
Muscle	12.5 ± 1.3	15.5 ± 1.6
Ovaries		18.1 ± 1.1
Pancreas	268 ± 137	299 ± 152
Red Marrow	12.6 ± 1.6	15.2 ± 1.8
Osteogenic Cells	18.1 ± 1.2	23.7 ± 1.6
Skin	9.3 ± 0.8	11.6 ± 1.0
Spleen	18.7 ± 2.2	23.4 ± 2.7
Testes	11.0 ± 0.5	
Thymus	12.2 ± 1.1	15.7 ± 1.4
Thyroid	11.2 ± 0.5	13.1 ± 0.6
Urinary Bladder Wall	41.3 ± 7.1	53.6 ± 9.9
Uterus		18.9 ± 1.3
Total Body	15.0 ± 3.3	18.8 ± 4.2
Effective doses	25.1 ± 5.3	30.8 ± 6.7

## Discussion

^18^F-FDG is the PET probe most commonly used for cancer diagnosis, staging, restaging, and assessment of therapy responses. However, the accumulation of this probe in inflamed lesions can lead to false positive diagnoses^1–3^. Therefore, efforts to further develop tumor-specific PET probes are important for increasing the effectiveness of cancer screening and treatment programs. In the present study, we demonstrated that the PET probe ^18^F-FIMP is highly specific for LAT1, with high accumulation in tumor tissue but not in inflamed lesions. T24 has higher expression of LAT1 than LNZ308 in tumor tissue (Fig. 1b). However, our final goal is to image brain tumors with ^18^F-FIMP. So, we selected LNZ308 brain tumor cell line in this study, and used T24 as a positive control when examining LAT1 expression analyses. We first examined PET imaging in a subcutaneous tumor model implanted with LNZ308, and are currently considering PET imaging in a brain tumor model.

We confirmed that ^18^F-FIMP is stable in aqueous 3.5% ascorbic acid. Starting with a radiochemical purity of 99.1%, this value decreased to 97.6% within 24 h after synthesis. For practical clinical and research applications, this high stability is advantageous as it allows for possible transportation of the synthesized ^18^F-FIMP to hospitals and laboratories that are remote from the place of synthesis.

Inflammation is an inseparable by-product in the pathophysiology of cancer. Inflammation is not only a side-effect of cancer treatments, such as radio- and chemotherapy, but also contributes to the development and progression of cancer. At the earliest stages of neoplastic progression, inflammation can promote the progression of incipient neoplasia into invasive cancers^13^. Furthermore, inflammation can significantly hinder the efficacy of diagnostic tests. The accumulation of ^18^F-FIMP in inflamed lesions was low in both animal models of inflammation (turpentine oil induced myositis model and collagen induced arthritis model); hence, suggesting that this probe may provide a more accurate approach to discriminate tumor tissues from inflamed tissues.

The efflux of radiolabeled amino acids and their metabolites from cells has been negatively correlated with their accumulation in tumors^14^. Most natural amino acid-derived PET probes, such as ^11^C-MET, are incorporated into the protein fraction, resulting in an increase in nonspecific accumulation in non-tumor tissues^15^. From our results, ^18^F-FIMP showed very high metabolic stability both under *in vitro* and *in vivo* conditions and is not incorporated into protein. These findings suggest that the high tumor accumulation value obtained using this tumor-specific PET probe maybe more reliable compared to that of other radiolabeled amino acids such as L-3-^18^F-fluoro-α-methyl tyrosine (FAMT)^9^, *O*-(2-^18^F-fluoroethyl)-L-tyrosine (FET)^16^, and *anti*-1-amino-3-^18^F-flurocyclobutane-1-carboxylic acid (FACBC)^8^. In order to demonstrate whether ^18^F-FIMP has superiority in tumor imaging, standard PET probes, such as ^18^F-FDG and ^11^C-MET, and ^18^F-FIMP need to be directly compared in the same cancer inoculation models by PET imaging studies.

An accurate estimation of radiation exposure is indispensable for defining a safe clinical PET study protocol. According to the biodistribution data from our PET imaging studies, we were able to estimate the expected mean absorbed dose of ^18^F-FIMP in humans. The effective doses of ^18^F-FIMP were determined to be 25.1 ± 5.3 and 30.8 ± 6.7 μSv/MBq for males and females, respectively. These doses are comparable or slightly higher than that of ^18^F-FDG (19.0 μSv/MBq for male) and ^18^F-fluroethyltyrosine (16.0 μSv/MBq for male)^17^ but still indicate that ^18^F-FIMP is a safe PET probe for use in humans.

However, additional studies are required to evaluate further applications of ^18^F-FIMP in humans. For example, additional comparisons between ^18^F-FIMP and other LAT1-specific PET imaging probes would provide a valuable assessment of its potential as a cancer diagnosis tool.

In this study, we developed a tumor imaging PET probe with a high affinity for LAT1. PET imaging studies revealed that ^18^F-FIMP accumulated in LAT1-positive tumor tissue, but not in inflamed lesions. This markedly high discrimination between tumors and inflamed lesions is important for effective diagnosis and treatment. Hence, ^18^F-FIMP may have advantages over existing PET imaging probes, such as ^18^F-FDG and ^11^C-MET.

## Methods

### Radiosynthesis of ^18^F-FIMP

Radiochemical syntheses were carried out in a hot cell, using a computer controlled automated radiochemical synthesis unit (Sumitomo Heavy Industries, Ltd., Tokyo, Japan). ^18^F-fluoride was produced *via* the ^18^O (p,n) ^18^F nuclear reaction, by irradiation of a 98% ^18^O-enriched water target with a 12 MeV proton beam at the HM-12S cyclotron (Sumitomo Heavy Industries, Ltd.). The aqueous solution containing ^18^F-fluoride ion was passed through Sep-Pak Accell Plus QMA Plus Light Cartridge (Waters Corporation, Milford, MA) and adsorbed ^18^F-fluoride ions were eluted with acetonitrile (0.7 mL), Kryptofix 2.2.2 (14 mg), and aqueous potassium carbonate (0.2 mL of 0.21 mol/L). The solvent was removed by azeotropic distillation with acetonitrile under He atmosphere.

**Figure 7:**
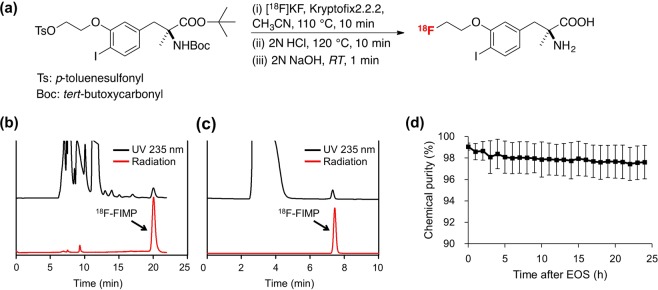
Synthesis of ^18^F-FIMP and quality confirmation. **(a)** Synthesis of ^18^F-FIMP via ^18^F-fluorination, deprotection, and neutralization. **(b)** Semi-preparative HPLC chromatogram of ^18^F-FIMP. **(c)** Radio-HPLC analysis of ^18^F-FIMP purity. **(d)** Stability of ^18^F-FIMP in saline containing 3.5% ascorbic acid over time after end of synthesis (EOS). Data are presented as mean ± SD (n = 4).

^18^F-FIMP was prepared *via* a three-step reaction that consisted of ^l8^F-fluorination of a tosyl precursor, deprotection, and neutralization (Fig. 7a). For example, the tosyl precursor (10 mg, 14.8 μmol) in acetonitrile (500 μL) was reacted with ^18^F-fluoride ions at 110 °C for 10 min. The fluorinated product was deprotected in 2 N HCl (500 μL) at 120 °C for 10 min, then the solution was neutralized by adding 2 N sodium hydroxide (500 μL). The solution was adjusted to pH 6 by addition of acetic acid (100 µL) and H_2_O (500 µL) and allowed to equilibrate at room temperature for 1 min. The crude ^18^F-FIMP was purified by reverse-phase HPLC (column: COSMOSIL 5C18-AR-II packed, 20 mm × 250 mm; eluent, a 45:55 methanol/20 mM phosphate buffer (pH 2.5); flow rate, 10 mL/min; detection, UV absorption by 235 nm and gamma-ray detector) (Fig. 7b). After radiopharmaceutical formulation using sterile water (1 mL) and a 25% aqueous solution of ascorbic acid (500 μL), the ^18^F-FIMP purity was measured using analytical HPLC, which was performed using a 4.6 mm × 250 mm column and elution with 50:50 methanol/20 mM phosphate buffer (pH 2.5; Fig. 7c). The purity (radiochemical purity, 98.6 ± 1.1%; the chemical purity, >99%; specific activity, 122 ± 3 GBq/μmol; n = 8) was determined sufficient for application in subsequent animal PET studies.

^18^F-FIMP stability was estimated under *in vitro* conditions. ^18^F-FIMP showed high stability (97.6%) standing at room temperature for 24 h after the synthesis (Fig. 7d).

### Radiosynthesis of ^18^F-FET, ^11^C-methionine, and ^18^F-FDG

^18^F-labeled fluoroethyl-L-tyrosine (^18^F-FET) was produced by phase transfer–mediated nucleophilic ^18^F fluorination of N-trityl-O-(2-tosyloxyethyl)-L-tyrosine-tert-butyl ester and subsequent deprotection with a radiochemical purity of >99.5% determined by HPLC. ^11^C-Methionine (^11^C-MET) was synthesized as described previously^18^, with a radiochemical purity of >99.5% determined by HPLC. ^18^F-FDG for human diagnostic grade was provided by the Institute of Biomedical Research and Innovation (IBRI) hospital, Kobe, Japan.

### Cell culture

The LNZ308 human glioblastoma cell line was a kind gift from Prof. Motoo Nagane of Department of Neurosurgery, Kyorin University, Japan. T24 (RCB0431) human bladder transitional-cell carcinoma and WI-38 (RCB0702) human lung normal fibroblast cells were obtained from the RIKEN BRC through the National Bio-Resource Project of the MEXT/AMED, Japan. LNZ308 and WI-38 cells were cultured in Dulbecco’s modified Eagle’s medium (Nacalai Tesque, Inc., Kyoto, Japan) supplemented with 10% fetal bovine serum (FBS), 100 units/mL penicillin, and 100 μg/mL streptomycin. T24 cells were cultured in McCoy’s 5 A (Modified) medium (Thermo Fisher Scientific K.K., Tokyo, Japan) supplemented with 10% FBS, 100 units/mL penicillin, and 100 μg/mL streptomycin.

### Subcutaneous tumor xenograft and inflammation models

All animal experimental protocols were approved by the Animal Care and Use Committee of RIKEN and performed in accordance with the National Institutes of Health Principles of Laboratory Animal Care (Approved No. MAH28-02). All applicable institutional and/or national guidelines for the care and use of animals were followed.

LAT1-positive human glioblastoma (LNZ308) cells were inoculated into the right forepaws of female BALB/cAJcl-nu/nu nude mice (CLEA Japan, Inc., Tokyo, Japan) *via* subcutaneous injection of 5 × 10^6^ cells in 100 μL phosphate buffered saline (PBS). Tumor growth was monitored twice weekly using a caliper. Acute-phase inflammation was induced by subcutaneous injection of 50 μL turpentine oil into the left forepaw of tumor-bearing mice 72 h before PET imaging^19^. We also used collagen-induced arthritis (CIA) model mice for evaluation of developed radiolabeled probes (see Supplementary Methods).

### PET data acquisition

Mice were fasted for 14 h before ^18^F-FDG administration. ^18^F-FIMP and ^11^C-MET-PET were administered to unfasted mice. All mice were anesthetized with 1.5% isoflurane and placed on the bed of a microPET Focus 220 scanner (Siemens Medical Solutions USA, Inc., Knoxville, TN). Radiolabeled probes were dissolved in saline (0.1 mL) and administered via a cannula inserted into the tail vein. Emission data were acquired for 90 min after administration using a 3-dimensional (3D) list-mode method for ^18^F-FIMP and ^11^C-MET, and for 45 min from 55 min after administration using a 3D list-mode method for ^18^F-FDG. Data were reconstructed using 2-dimensional filtered back projection (FBP) for quantification and a 2-dimensional ordered subset expectation maximization (OS-EM) algorithm for region of interest (ROI) definition. For ROI definition and further analysis, summed images (0–90 or 55–100 min post injection) were reconstructed. ROIs were drawn on several areas of tumor, muscle, and inflamed tissues. Regional uptake of radioactivity in organs were decay-corrected based on injection times and expressed as the standardized uptake value (SUV), where SUV = tissue radioactivity concentration (MBq/cm^3^)/injected radioactivity (MBq) × body weight (g). Quantitative analysis of PET imaging data also represented as tumor-to-muscle and inflamed lesion-to-normal muscle ratios (TMR and INR, respectively). PET imaging was also performed with CIA mice using ^18^F-FIMP, ^11^C-MET and ^18^F-FDG.

After PET imaging, mice were sacrificed and their organs quickly removed and washed with saline. Blood samples were obtained immediately before dissection by cardiac puncture. Excised organs and blood samples were weighed and their radioactivity determined using a Wallac Wizard 1480 scintillation counter (PerkinElmer, Waltham, MA). Results were expressed as %injected dose per gram of tissue, TMR, tumor-to-blood ratio (TBR), and INR.

### Dosimetry analysis

Mean absorbed doses of ^18^F-FIMP (μSv/MBq) in humans were estimated on the basis of PET imaging data from mice (n = 4). The mean %ID/g values for mouse livers, kidneys, pancreases, urinary bladders, and remainder of the body were extrapolated to estimate expected uptake in organs for a 73 kg human adult male. The organ fractions of total body mass for mice (25 g), human males (73 kg), and human females (53 kg) required for this extrapolation were obtained from Hui *et al*.^20^ for mice and ICRP Publication 60^17^ for humans, respectively. Dosimetry estimations were calculated using the OLINDA/EXM version 1.1 software (Hermes Medical Solutions, Stockholm, Sweden) based on standard human male and female models^21^.

### Statistical analysis

Data are presented as mean ± standard deviation (SD). All statistical analyses were performed using Microsoft Excel 2010 version 14.0 (Microsoft, Redmond, WA) using Student’s t test. P-values less than 0.05 were considered significant.

## Supplementary information

Supplementary information
