# Supplementary material for: 18F-FIMP: a LAT1-specific PET probe for discrimination between tumor tissue and inflammation
Source: Sci Rep. 2019 Oct 31;9:15718. doi: 10.1038/s41598-019-52270-x (PMC6823354; doi:10.1038/s41598-019-52270-x)
Supplement: Supplementary file 1 — Supplementary information [file 41598_2019_52270_MOESM1_ESM.pdf]

Supplementary information

^{18}F-FIMP: a LAT1-specific PET probe for discrimination between tumor tissue and inflammation

Satoshi Nozaki, Yuka Nakatani, Aya Mawatari, Nina Shibata, William E. Hume, Emi Hayashinaka, Yasuhiro Wada, Hisashi Doi, and Yasuyoshi Watanabe[*]

Methods

Expression of human LAT1 and CD98 in tumor cell lines and tumors

LAT1 is a predicted 12 transmembrane protein and is unique because it requires an additional single transmembrane protein, CD98, for its functional expression. Before PET imaging in mice, we evaluated LAT1 and CD98 expression in tumor cell lines and xenografts using a Western blot. LNZ308, T24, and WI-38 crude cell membrane fractions were prepared as described previously[1]. Cell pellets were suspended in homogenization buffer containing 50 mM Tris-HCl (pH 7.4), 150 mM NaCl, 5 mM ethylenediaminetetraacetic acid (EDTA), 1 mM phenylmethylsulfonyl fluoride (PMSF),

1 mM sodium orthovanadate (V) and 1 mM NaF, and homogenized by sonication. Homogenates were centrifuged at 1,000 × *g* at 4°C for 5 min, then the supernatants centrifuged at 450,000 × *g* at 4°C for 30 min and the membrane pellet was resuspended in resuspension buffer containing 50 mM Tris-HCl (pH 7.4), 150 mM NaCl, 1 mM EDTA, 1 mM PMSF, and 1% NP-40. Sample protein concentration was determined using the bicinchoninic acid (BCA) method. The protein sample was separated by SDS-PAGE using a 10-20% gradient polyacrylamide gel and separated proteins transferred electrophoretically to a Hybond-P PVDF transfer membrane (GE Healthcare, Chicago, IL). The membrane was pre-blocked in Bullet Blocking One blocking solution (Nacalai Tesque, Inc.) at room temperature for 1 h. The membrane was then incubated with the blocking solution containing a 1:10,000 dilution of rabbit anti-human LAT1 polyclonal antibody (TransGenic Inc., Fukuoka, Japan), 1:10,000 dilution of rabbit anti-human CD98 polyclonal antibody (Santa Cruz Biotechnology, Inc., Santa Cruz, CA), or 1:100,000 dilution of rabbit anti-human sodium potassium ATPase monoclonal antibody (Abcam Inc., Cambridge, UK). The membrane was treated with horseradish-peroxidase-conjugated anti-rabbit IgG (Sigma-Aldrich Co. LLC., St. Louis, MO) and developed using ECL Select Western Blotting Detection Reagent (GE Healthcare) before visualization under an LAS-3000 luminescent image analyzer

(Fujifilm Corporation, Tokyo, Japan).

Collagen-induced arthritis (CIA) model

CIA model mice were produced by Japan SLC, Inc., Hamamatsu, Japan. Briefly, eight-week-old male DBA/1J Jms Slc mice were subcutaneously injected with 0.025 mL of the emulsion containing 0.1 mg of bovine type II collagen in Freund's complete adjuvant into the basement of the auricle on day 0. An 0.025 mL booster injection of the same concentration was given on day 21 into the hip region close to the tail. Swelling of the hind paw was assessed by plethysmography immediately before PET imaging.

Metabolite analysis

In vitro screening of metabolic stability with liver microsomes (human, rat and mouse) was performed by BoZo Research Center Inc., Tokyo, Japan. Briefly, liver microsomes, enzyme cofactors, and test compound were mixed in an isotonic buffer. After the incubation period (0.5-1 h), an equal volume of an organic solvent (e.g. acetonitrile) was added to stop the reaction and extract the test compounds from the assay. The

organic extracts were then analyzed by LC-MS/MS, and metabolic stability expressed as the percentage of parent compound remaining.

Metabolites in the plasma, urine, liver, pancreas, and tumor tissues from tumor-bearing mice after injection of ^{18}F-FIMP were analyzed by thin-layer chromatography (TLC) in our laboratory. ^{18}F-FIMP (ca. 50 MBq per animal) was injected *via* a lateral tail vein into 8 to 10-week-old mice. Whole blood, urine, and tissues were collected at 90 min after injection. Blood was collected in heparinized tubes and centrifuged at 13,000 × *g*, at 4°C for 5 min, to separate blood cells from plasma. Tissues were homogenized with 2 volumes of ice-cold water by Polytron homogenizer. Plasma, urine and tissue homogenates (100 μL each) were mixed with acetonitrile (100 μL) and centrifuged at 5,800 × *g* for 5 min. The supernatant was applied to a silica gel 60 F_{254} TLC plate (Merck KGaA, Darmstadt, Germany) and developed with 1-butanol/acetic acid/PBS (4:1:2, pH 7.4). After drying and marking the solvent front and the origin with radioactivity, the TLC plates were put in contact with a phosphor imaging plate. The exposed images were read with a FLA-7000 phosphor imager and analyzed with Multi Gage, version 3.1 (both Fujifilm Corporation, Tokyo, Japan). The percentages of unchanged fractions in selected regions of interest were estimated by dividing the peak area of parent compound (^{18}F-FIMP) by the total area of all chromatographic peaks.

Protein incorporation assay

LNZ308 cells were seeded in 6-well dishes. After changing the medium, cells were incubated with or without 100 μg/mL cycloheximide for 30 min in a CO_2 incubator. ^{18}F-FIMP (37 kBq) or ^{11}C-MET (370 kBq) was added to the medium followed by incubations for another 60 min. After removal of the radioactive medium, cells were washed three times with ice-cold PBS, treated with 1 mL of trichloroacetic acid for 10 min on ice, and then the detached cells were resuspended and collected. The samples were centrifuged at 13,000 × *g*, 4°C for 10 min, and the radioactivity of the supernatant and precipitate measured by using a Wallac Wizard 1480 scintillation counter (PerkinElmer, Waltham, MA). Protein incorporation was calculated as the percentage of acid-precipitated radioactivity.

Reference

1 Khunweeraphong, N. *et al.* Establishment of stable cell lines with high expression of heterodimers of human 4F2hc and human amino acid transporter LAT1 or LAT2 and delineation of their differential interaction with alpha-alkyl moieties. *J Pharmacol Sci* **119**, 368-380 (2012).

Supplementary Fig. S1

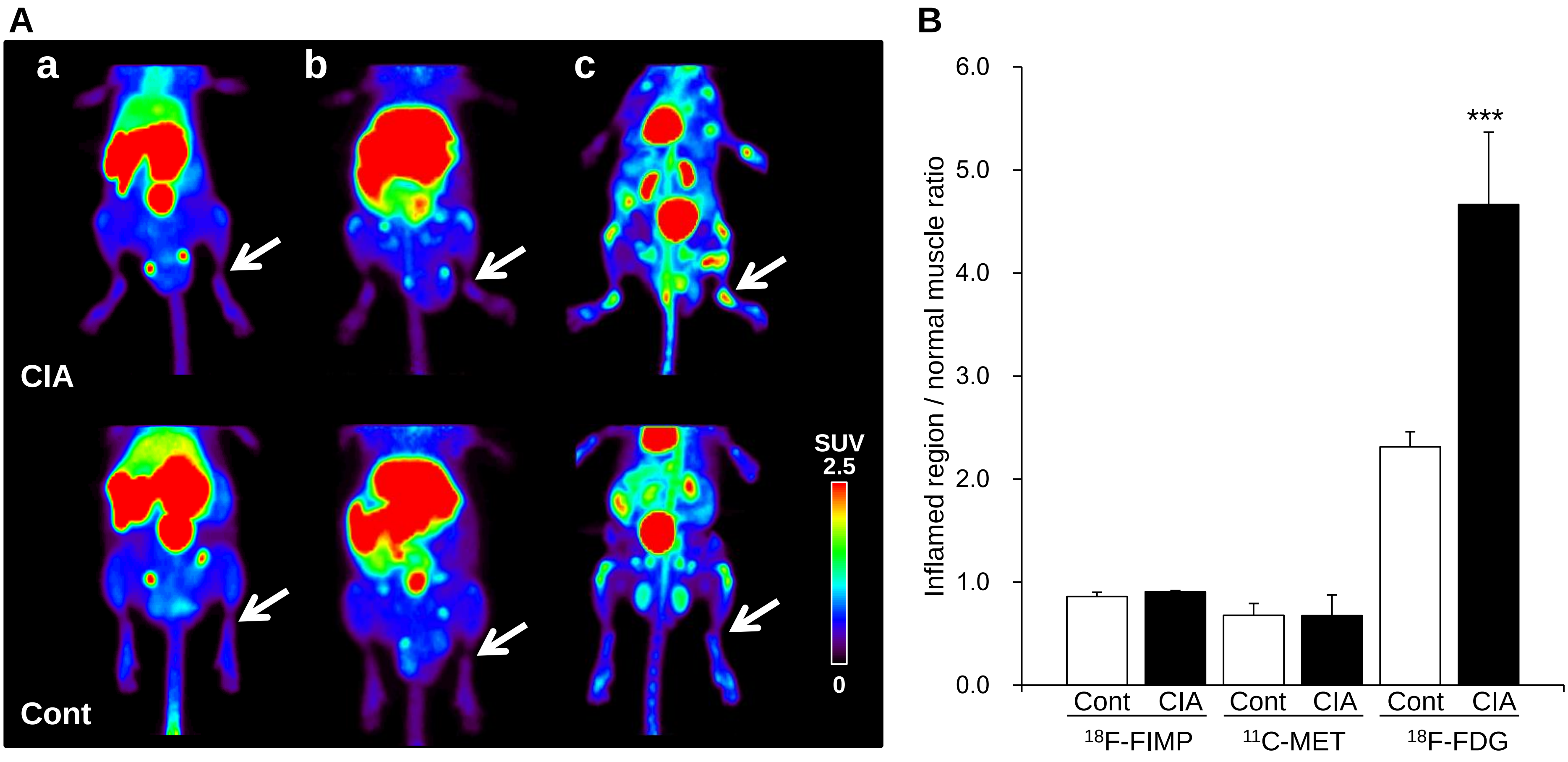

Supplementary Fig. S1 PET probe accumulation in collagen-induced arthritis model mice
Male DBA/1J Jms Slc mice were immunized via subcutaneous injection with bovine type II collagen in Freund's complete adjuvant. Swelling of the hind paw (arrow) was assessed just before PET imaging by plethysmography. PET data were acquired using microPET Focus220 for 90 min after injection of ^{18}F-FIMP (A-a) and ^{11}C-MET (A-b). Only ^{18}F-FDG-PET data (A-c) were acquired from 45 to 90 min after injection. PET imaging data were quantitatively analyzed and shown as the (B) inflamed region to normal muscle ratio. ***P < 0.001, compared with control groups. Data are presented as mean ± SD (N=4).

Supplementary Fig. S2

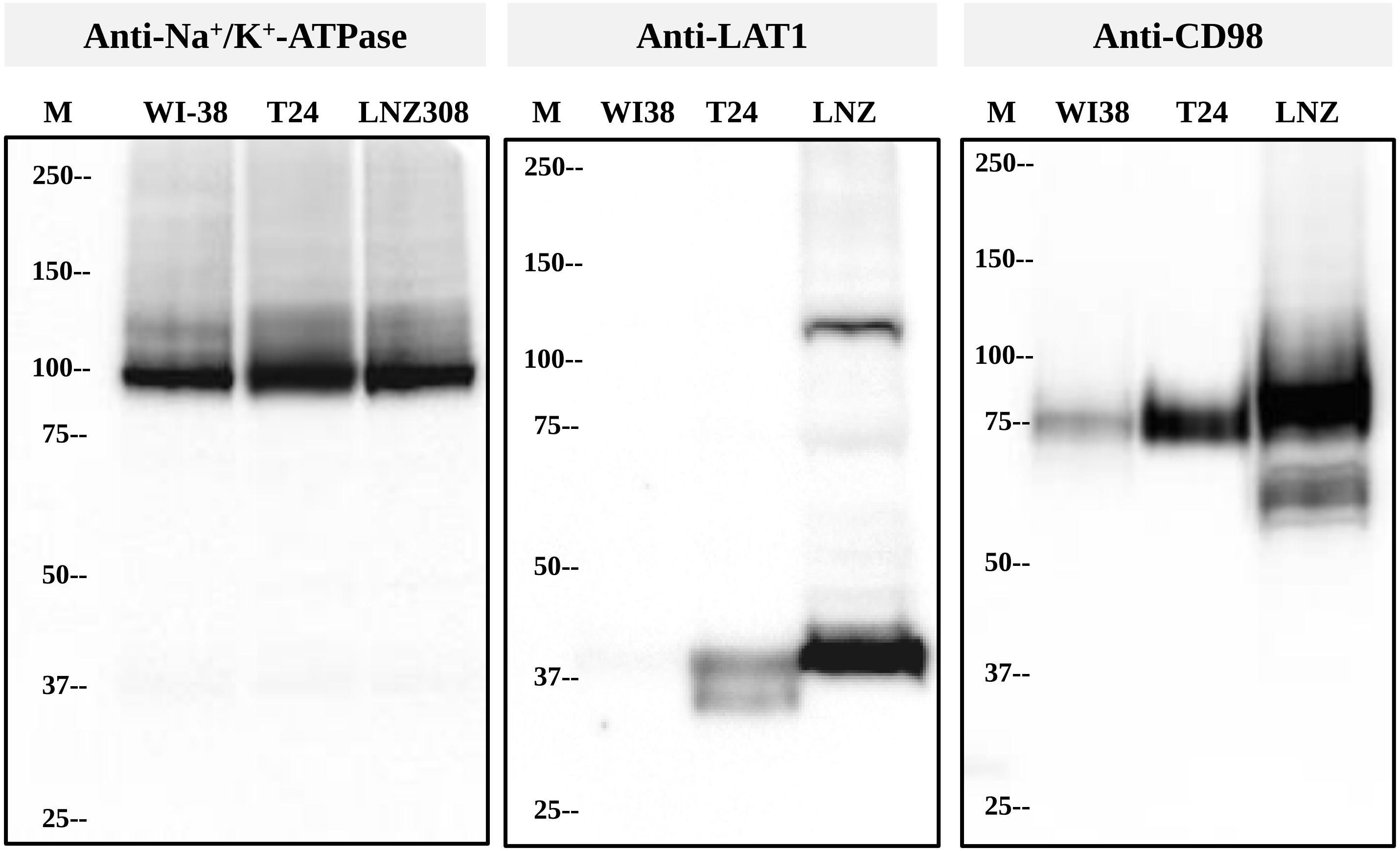

M: Size marker (kDa)
WI-38: WI-38 cells crude membrane protein (20 µg protein/lane)
T24: T24 cells crude membrane protein (20 µg protein/lane)
LNZ308: LNZ308 cells crude membrane protein (20 µg protein/lane)

Supplementary Fig. S3

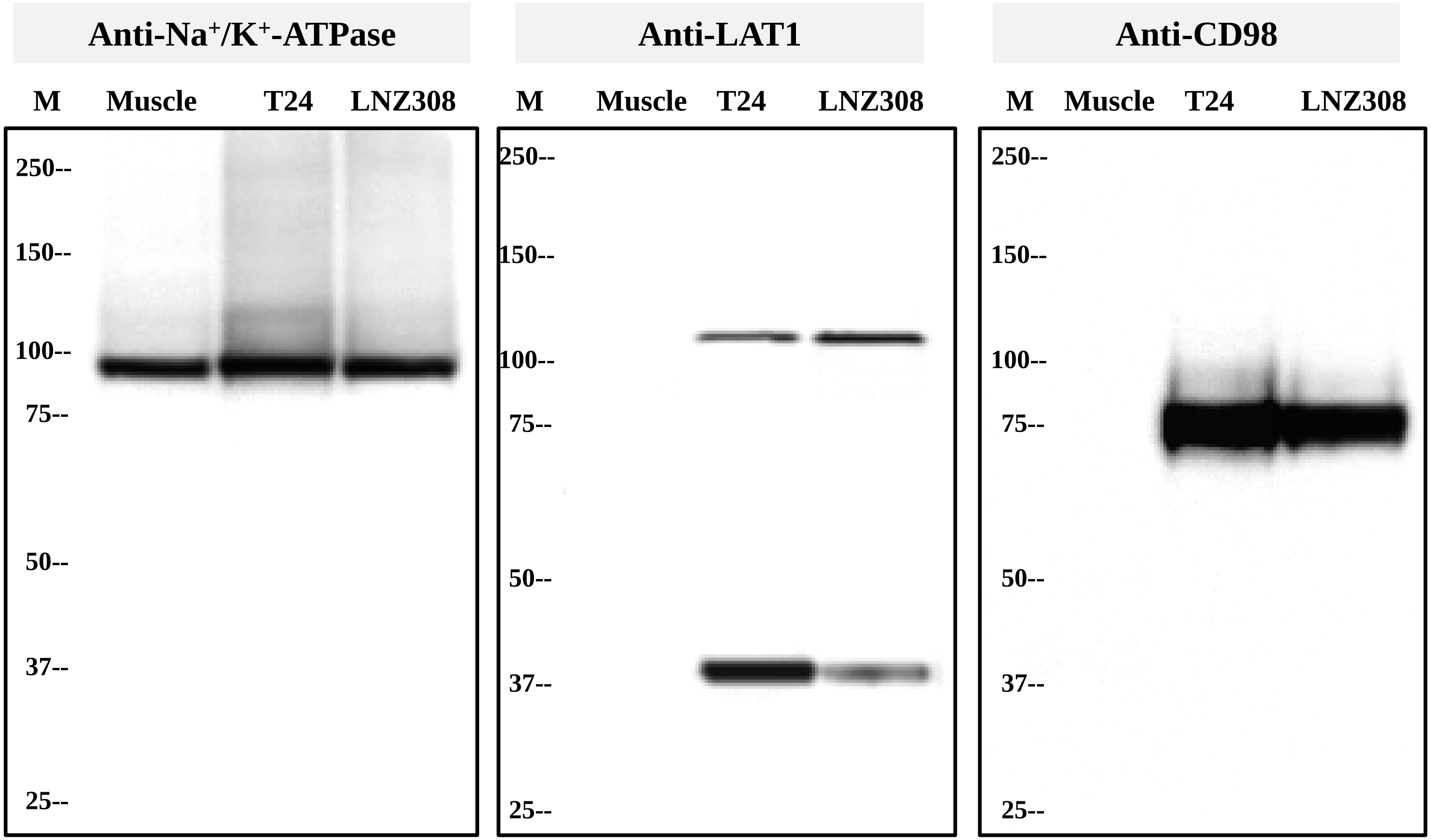

M: Size marker (kDa)
Muscle: Muscle tissue crude membrane protein (40 µg protein/lane)
T24: T24 tumor crude membrane protein (40 µg protein/lane)
LNZ308: LNZ308 tumor crude membrane protein (40 µg protein/lane)
